# Neurometabolite alterations in Gulf War Illness: a whole-brain magnetic resonance spectroscopy study

**DOI:** 10.1007/s00221-025-07174-w

**Published:** 2025-10-25

**Authors:** Chloe Jones, Olivia Haskin, Jarred Younger

**Affiliations:** Department of Psychology, The University of Alabama at Birmingham, Birmingham, AL 35233 USA

**Keywords:** Magnetic resonance spectroscopy, Gulf War Syndrome, Neuroinflammation, Choline, NAA

## Abstract

Gulf War Illness (GWI) affects approximately 30% of veterans who served in the 1991 Persian Gulf War and is characterized by chronic pain and fatigue, as well as cognitive, mood, gastrointestinal, and respiratory symptoms. Animal research has suggested that GWI is caused by a combination of neurotoxicants such as nerve gas, anti-nerve agent pills, and pesticides, though a definitive pathophysiological model has not been established. In this human observational study, 20 veterans with GWI and 20 healthy Gulf War veterans (HV) underwent whole-brain magnetic resonance spectroscopy to non-invasively measure several metabolites associated with neuroinflammation. Veterans also completed an arterial spin labeling scan to assess cerebral perfusion. Compared to HV, veterans with GWI demonstrated widespread decreases in brain choline, *N*-acetylaspartate, and creatine, and regional elevations in lactate and brain temperature. No group difference was observed in cerebral perfusion. Exploratory analyses revealed brain metabolites were associated with self-reported neurotoxicant exposures in theater. These findings support a role of cholinergic alterations and neuroinflammatory processes in GWI.

*Trial registration*: The study was registered in ClinicalTrials.gov, ID NCT04638998. Registered November 16, 2020, https://clinicaltrials.gov/study/NCT04638998.

**Supplementary Information:**

The online version contains supplementary material available at 10.1007/s00221-025-07174-w.

## Background

Gulf War Illness (GWI) refers to the constellation of respiratory, dermatological, gastrointestinal, fatigue, pain, mood, and neurological symptoms observed in approximately 1/3 of the nearly 700,000 US veterans deployed to the 1990–1991 Persian Gulf War (Fukuda et al. 1998; Steele 2000). The Department of Defense 2020 Gulf War Illness Landscape Report (Department of Defense Gulf War Illness Research Program, March 2020) concluded that over the last 25 years, few veterans with GWI have experienced symptom improvement and many are now developing age-related comorbidities. As Gulf War veterans are at greater risk of developing chronic age-related conditions (Zundel et al. 2019), the need grows greater to identify treatments to improve quality of life. There are currently no approved treatments or biomarkers for GWI. A major obstacle to developing treatments that target core pathophysiology has been the lack of biomarkers and established mechanisms.

While deployed, servicemembers were exposed to numerous toxins including chemical pesticides, depleted uranium, pyridostigmine bromide (PB), sarin nerve gas, solvents, environmental pollutants, oil well fires, dust storms, endemic diseases, anthrax vaccines, and botulinum toxoid vaccines (Fulco et al. 2000). Of these exposures, research has focused on cholinergic compounds present in the region–sarin and cyclosarin, PB pills, and chemical pesticides. Animal models have demonstrated that even at low doses, sarin can impact immune responses (Kalra et al. 2002) and lower acetylcholinesterase levels in the brain despite having no behavioral acute effects (Henderson et al. 2002). Although no conclusive evidence has connected the use of PB pills to the development of GWI, a combination of PB and insecticides has long been a focus of research on the illness (Golomb 2008; White et al. 2016).

It has also been proposed that although the development of GWI may be initiated by acetylcholinesterase inhibitors, it is not because of their cholinergic effects, but because of organophosphorylation of non-cholinergic targets resulting in proinflammatory processes (Michalovicz et al. 2020). The majority of literature supporting neuroimmune dysfunction in GWI has been preclinical, however, a positron emission tomography (PET) study in 2020 provided the first in vivo evidence of neuroinflammation in GWI (Alshelh et al. 2020). Compared to healthy Gulf War veterans and healthy civilian controls, veterans with GWI showed greater signal in several brain regions with the radioligand [^11^C]PBR28, which binds to the 18 kDa translocator protein (TSPO) that is upregulated in activated microglia.

Although acetylcholine cannot be directly quantified in humans, levels of choline-containing compounds in the brain (i.e., choline, phosphocholine, and gylcerophosphocoline) can be estimated through proton magnetic resonance spectroscopy (MRS) (Miller et al. 1996). The choline signal in MRS is thought to provide a reliable estimate of acetylcholine due to their linear relationship (Mineur and Picciotto 2023). The correlation between the MRS choline signal and levels of acetylcholine in the brains of rodents ranges from 0.82 to 0.85 (Wang et al. 2008).

Only a few human MRS neuroimaging studies have evaluated GWI, and these studies primarily measured *N*-acetylaspartate (NAA) as a marker of neuronal integrity or density. Lower NAA referenced to creatine (NAA/Cr) in GWI has been seen in the pons and basal ganglia (Haley et al. 2000; Meyerhoff et al. 2001), but this has not been replicated in larger samples (Weiner et al. 2011). These single-voxel MRS studies were conducted on less-powerful 1.5 T scanners and limited to the regions selected a priori. 3D echo-planar spectroscopic MRS techniques have since been developed that allow for efficient collection of metabolites across the whole brain.

In addition to choline compounds, whole-brain MRS can also provide spectra for other metabolites that serve as neuroinflammatory markers. Increased lactate represents greater energy demands of activated microglia and anerobic metabolism, while increased choline and myo-inositol reflect glial cell density and turnover (Quarantelli 2015; Albrecht et al. 2016). Chronic neuroinflammation is also associated with decreased NAA on MRS due to decreased neuronal integrity (Chang et al. 2013; Wang et al. 2015). Furthermore, brain temperature can be indirectly measured with MRS by calculating the difference between temperature-sensitive water and temperature insensitive metabolite resonance peaks (Cady et al. 2011). Elevated brain temperature may reflect increased metabolism and cerebral blood flow (Yulug et al. 2023), but also central nervous system inflammation (Plank et al. 2022), or inefficient cooling mechanisms, such as reduced heat exchange with arterial blood due to poor perfusion (Zhu et al. 2006; Wang et al. 2014). This human observational study is the first to use whole-brain MRS imaging in GWI. By comparing brain metabolites between veterans with GWI and healthy Gulf War veterans (HV), we aim to investigate the role of neuroimmune and cholinergic dysfunction in GWI.

## Methods

### Participants

Participants were recruited via social media advertisements by High Level Marketing (Birmingham, AL), laboratory social media postings, and flyers around the University of Alabama at Birmingham (UAB) campus. All advertisements were approved by the UAB Institutional Review Board (IRB). Veterans who had previously participated in our laboratory studies and who stated they were interested in future studies were directly contacted and notified of the enrollment opportunity. Participants were reimbursed $150 for completing the study.

#### Inclusionary criteria

Eligibility was assessed via phone screening and Qualtrics survey (Qualtrics, Provo, UT). Male veterans of the US armed forces ages 46–65 who were deployed to the Persian Gulf Region between August 1990–July 1991 were recruited. Participants were considered to have GWI if they met the Kansas case inclusionary criteria (Steele 2000), which requires at least moderate severity on 3 of 6 chronic symptom domains. These domains include fatigue/sleep, pain, neurologic/cognitive/mood, gastrointestinal, respiratory, and skin. Symptoms must have emerged during or after Persian Gulf deployment to be considered positive. Symptoms were rated as none, trivial, mild, moderate, or severe within the last 6 months. HV were defined as Gulf War veterans who did not meet GWI criteria, and who also did not report significant daily fatigue or pain (i.e., rated fatigue and body pain as mild or less on Kansas case criteria).

#### Exclusionary criteria

Potential participants were excluded for concurrent participation in another clinical trial, use of opioids, stimulants, or sedatives within the last 6 months, history of cancer, moderate or severe traumatic brain injury, stroke, major cardiovascular illness, blood or blood clotting disorder, HIV, Hepatitis C, thyroid disorder, or uncontrolled diabetes (i.e., A1C ≥ 9%). Potential participants were deferred or excluded for recent vaccination (within the last 4 weeks), recent illness/infection, surgery, or injury (within the last 3 months), concussion, whiplash injury, other mild head trauma within last 3 months, or involvement in ongoing litigation related to GWI symptoms. Participants were administered the Hospital Anxiety and Depression Scale (HADS), and participants were excluded if they endorsed severe depression as defined by a depression subscale score of greater than 15. In accordance with the Kansas GWI case definition exclusion criteria, participants could not be considered a positive GWI case if they had a history of stroke, lupus, multiple sclerosis, melanoma, cancer, liver disease, alcohol or drug dependence resulting in hospitalization, depression or posttraumatic stress disorder (PTSD) resulting in hospitalization, schizophrenia, or bipolar disorder. Participants were also screened for MRI contraindications.

### Study protocol

This study was registered in ClinicalTrials.gov (ID: NCT04638998). The protocol was approved by the UAB IRB. Eligible participants were invited to complete the single-visit study protocol at UAB. All participants provided informed consent prior to participation. After providing consent, participants completed self-report questionnaires and underwent MRI brain scans at the Civitan International Neuroimaging Laboratory (CINL). Questionnaires included demographic information, the Hospital Anxiety and Depression Scale (HADS) (Zigmond and Snaith 1983), Kansas Gulf War Experiences and Exposure Questionnaire (Steele 2000), Patient Health Questionnaire (PHQ-9) (Kroenke et al. 2001), Fatigue Severity Scale (FSS) (Krupp et al. 1989), Posttraumatic Stress Disorder Checklist for DSM-5 with Criterion A (PCL-5) (Weathers et al. 2013), Center for Disease Control Gulf War Illness Case Criteria (Fukuda et al. 1998), and the Brief Pain Inventory—Short Form (BPI) (Cleeland and Ryan 1991, 1994). Prior to the brain scan, all participants completed and signed an MRI safety screening form, were changed into scrubs or hospital gown, and were screened for metal with a metal detector. Participants’ body temperature was collected in both ears before and after imaging.

### MRI

#### Structural image

MRI data were collected at the UAB Civitan International Neuroimaging Laboratory (CINL) using a Siemens Magnetom Prisma 3 Tesla scanner. Participants’ heads were cushioned in a 20-channel head/neck coil for stability, and they were provided with earplugs for hearing protection. A T1-weighted (T1w) structural image sequence (magnetization-prepared rapid gradient echo, MPRAGE) was acquired with the parameters: TR = 2.0 s, TE = 2.51 ms; 208 slices; flip angle = 8°; isotropic 0.9 mm voxel; TI = 901 ms; FOV = 230 × 230 mm; total acquisition time of 5 min.

#### Spectroscopy

Whole brain MRS data were collected with fast 3D echoplanar spectroscopic imaging (EPSI): TR_1_ = 1550 ms; TR_2_ = 511 ms; TE = 17.6 ms; flip angle = 71°; 5.6 × 6.5 × 10.0 mm voxel; TI = 198 ms; FOV 280 mm × 280 mm; generalized auto calibrating partially parallel acquisitions (GRAPPA) factor = 1.3; total acquisition time of 18 min.

Volumetric EPSI data processing was performed with the automated Metabolite Imaging and Data Analysis System (MIDAS) as described in Maudsley et al. (2009). Processing steps included spatial reconstruction, B0 inhomogeneity correction, spatial registration, lipid suppression, spectral fitting, quality assessments, signal normalization, and tissue segmentation. Voxels were excluded due to a fitted linewidth of greater than 13 Hz, a Cramer Rao lower bound of less than 40% for creatine fitting, a volume of greater than 30% cerebrospinal fluid (CSF), or values that were 2.5 times greater than the standard deviation within the ppm range of 3.6–1.7 (Maudsley et al. 2010). An amplitude-weighted combination of NAA, creatine, and choline was used as the reference for temperature deviations due to their insensitivity to temperature and susceptibility effects (Cady et al. 2011; Maudsley et al. 2017). Metabolite and temperature maps were co-registered to structural images and transformed to standard Montreal Neurological Institute (MNI) space. The Automated Anatomical Labeling (AAL) atlas (Tzourio-Mazoyer et al. 2002) was used to define 47 regions of interest (ROIs) in participant space using the PRANA module in MIDAS. The MINT module in MIDAS was used to integrate spectra from voxels in each ROI for a higher signal-to-noise ratio and more accurate fitting than for individual voxels.

#### Metabolite expression

Creatine is often used as an internal reference to express metabolite concentrations as it is assumed to be stable in most pathological conditions, but this practice has been debated (Li et al. 2003; Rae 2014; Rackayova et al. 2017). Creatine’s distribution across the brain can vary based on activity and blood supply (Rae 2014), and can also be affected by age (Haga et al. 2009; Lind et al. 2020), neuroinflammatory processes (Chang et al. 2013; Eisele et al. 2020), and psychiatric and neurological conditions (Malhi et al. 2002; Öngür et al. 2009; Yue et al. 2012; Wan et al. 2022). This variability can make interpretation of metabolite ratios referenced to creatine difficult to interpret. Alternatively, tissue water has been used as an internal reference, and has shown similar or better variance and reliability to creatine as a reference (Minati et al. 2010; Mikkelsen et al. 2019). As water content can also change in pathological states such as multiple sclerosis (Vargas et al. 2015), metabolites referenced to tissue water have their own interpretability challenges. To assess the most suitable reference for metabolite expressions in the present study, regional creatine and regional tissue water were compared between groups.

#### Arterial spin labeling

Pulsed ASL data were acquired with a commercially available Siemens product Proximal Inversion with Control of Off-Resonance Effects (PICORE) with Quantitative Imaging of Perfusion Using a Single Subtraction, second version, with thin-slice TI1 periodic saturation (Q2TIPS) (Luh et al. 1999). TR = 2500 ms; TE = 12.0 ms; TI = 1800 ms; bolus duration = 700 ms; 12 slices; 8.0 mm slice thickness; FOV = 256 × 256 mm; matrix 64 × 64; voxel size = 4.0 × 4.0 × 8.0; total acquisition time of 5 min. Sixty label-control image pairs and one calibration M0 image were acquired.

ASL data were processed with Bayesian Inference for Arterial Spin Labeling (FSL-BASIL, FMRIB Software Library v6.0, Oxford, UK) toolbox (Chappell et al. 2023) to obtain perfusion maps. Motion correction was performed with the MCFLIRT tool, and structural data was preprocessed using FSL’s Anatomical Processing Script to obtain partial volume estimates and tissue specific perfusion estimates. Voxels were defined as being grey matter or white matter by 80% and 90% partial volume, respectively. Kinetic model inversion and calibration were performed on the averaged subtracted label-control images to obtain absolute values of perfusion in ml/100 g/min. Cerebral spinal fluid was selected as the tissue reference. All parameters were set in accordance with the white paper (Alsop et al. 2015).

### Statistical analyses

Mean age and body mass index (BMI) were compared between groups with independent samples *t*-tests. Pearson Chi-square contingency tests were used to compare groups on the factors of handedness, ethnicity, household income, education, employment status, marital status, and nicotine use. If greater than 20% of the expected cell counts were less than 5, Fisher’s exact test was used. Reported Gulf War exposures from the Kansas Experiences and Exposure Questionnaire and scores on symptom questionnaires were treated as ordinal and compared between groups with Mann–Whitney U tests. Metabolites and tissue water for each of the 47 ROIs from MRSI scans were compared between groups with independent samples *t*-tests. Due to the high number of comparisons performed, a false discovery rate of α = 5% was used to minimize the proportion of false positive findings. Adjusted *p* values for each metabolite were calculated used the Benjamini–Hochberg procedure (Benjamini and Hochberg 1995). Mean grey matter perfusion was compared between groups with an independent samples *t*-test.

To further assess the impact of demographic variables on the results, a MANCOVA was performed with average brain metabolites as outcome variables, group as a fixed factor, and age, BMI, ethnicity, and education as covariates, entered simultaneously. Post-hoc pairwise comparisons with Bonferroni corrections were used to follow-up significant group differences.

Exploratory analyses assessing the relationship between Gulf War exposures and brain metabolites were performed with Spearman’s ranked correlations, where the reported frequency of each exposure was maintained in its original form as ordinal. For these exploratory analyses, to decrease the number of comparisons, spectra from the cortical lobes and cerebellum were averaged together for each metabolite to obtain a “whole-brain” metabolite average. To explore the potential confound of baseline PTSD group differences on NAA group differences, a sensitivity analysis was performed on a subsample that did not have significant differences in PCL-5 scores. To screen for the potential confound of anticholinergic medications on the MRS choline signal, all participants’ active medications were scored for possible anticholinergic effects using the Anticholinergic Cognitive Burden (ACB) Scale (Campbell et al. 2013). Medications were scored from zero to three for evidence of anticholinergic activity and all scores were summed to create an ACB score for each participant. Brain choline *t-*tests were re-run on a subsample with equivalent ACB score distributions. Exploratory nonparametric correlations between symptom questionnaires and brain metabolites controlling for age and BMI can be found in the Supplementary Materials (S3).

## Results

### Demographic information

Forty-seven veterans who were eligible for the study completed self-report questionnaires. Demographic information for the sample is displayed in Table 1. Groups were not significantly different on the variables of age (*p* = 0.63) or BMI (*p* = 0.87). Pearson Chi-Square contingency tests indicated that groups did not significantly vary on handedness, ethnicity, income, education, employment status, marital status, or nicotine use (Table 1). The GWI group reported greater fatigue, pain severity, pain interference, anxiety, depression, and posttraumatic stress symptoms than HV as can be seen in Table 2.

**Table 1:** Demographic characteristics

	HV	GWI	Statistic
	Mean		*t*
Age	55.19	54.73	0.48
BMI	29.02	29.52	− 0.36

	Count		χ^2^
Handedness			
Right	17	25	2.67
Left	4	1	
Hispanic			
No	21	24	n/a
Yes	0	0	
Ethnicity			
American Indian/Alaskan Native	1	1	1.77
Asian	0	1	
Caucasian	19	21	
Pacific Islander	0	1	
African American	1	2	
Income			
< $20,000	0	1	4.82
< $30,000	1	2	
< $40,000	0	0	
< $50,000	0	1	
< $60,000	1	1	
< $70,000	4	2	
< $80,000	0	2	
≥ $80,000	15	15	
Education			
Less than high school degree	0	0	6.77
High school degree	0	1	
Some college	3	7	
Associate degree	3	6	
Bachelor’s degree	10	5	
Master’s degree	4	6	
Professional degree	1	0	
Doctoral degree	0	1	
Marital status			
Never married	0	1	1.02
Married	16	18	
Divorced	5	7	
Widowed	0	0	
Employment			
Part-time	3	1	5.86
Full-time	16	19	
Retired	2	1	
Disabled	0	3	
Nicotine use			
No	15	23	1.99
Yes	6	3	

**Table 2:** Self-report questionnaires

Scale	HV	GWI	Statistic
	Mean	Mean	*U*
HADS anxiety subscale	4.84	9.13	342.00*
HADS depression subscale	3.47	7.21	347.00*
PTSD checklist for DSM-5	11.38	33.04	412.50**
Patient health questionnaire-9	2.90	10.62	486.50**
Fatigue severity score	2.57	4.71	466.50**
Brief pain inventory severity score	1.11	3.98	452.00**
Brief Pain inventory interference score	0.49	3.84	457.00**

*p < 0.01

**p < 0.001

### MRSI

Four participants who were enrolled did not complete the MRI brain scan due to claustrophobia upon entering the scanner or inability to fit in the scanner. A total of 43 participants completed the MRI brain scan. Three scans were excluded from analyses due to motion artifacts leaving 20 HV and 20 GWI datasets. Significant group differences for each metabolite are listed below; all findings including FDR-adjusted* p* values can be found in Supplementary Table 1 (S1).

#### Metabolite references

Total normalized creatine for reach region was compared between groups and was significantly different in 13 out of 47 regions (Table A11). Calibrated tissue water was not significantly different between groups for any of 47 brain regions tested (Table A13). Due to its consistency between groups, tissue water was selected as the most appropriate reference for metabolite analysis. All metabolites are expressed in institutional units. Findings for metabolites referenced to creatine (i.e., Lac/Cr, Cho/Cr, NAA/Cr, and MI/Cr) can be found in the Supplementary Materials (S2).

#### Creatine

Creatine normalized to water (Cr/H_2_O) was significantly lower in GWI in right precentral gyrus (*p* = 0.050), right mid cingulate (*p* = 0.049), left hippocampus (*p* = 0.040), left calcarine fissure (*p* = 0.005), right cuneus (*p* = 0.021), left cuneus (*p* = 0.016), left lingual gyrus (*p* = 0.022), left postcentral gyrus (*p* = 0.012), left paracentral lobule (*p* = 0.040), left globus pallidus (*p* = 0.044), left thalamus (*p* = 0.037), right temporal gyrus (*p* = 0.030), and left temporal gyrus (*p* = 0.040).

#### Choline

**Fig. 1:**
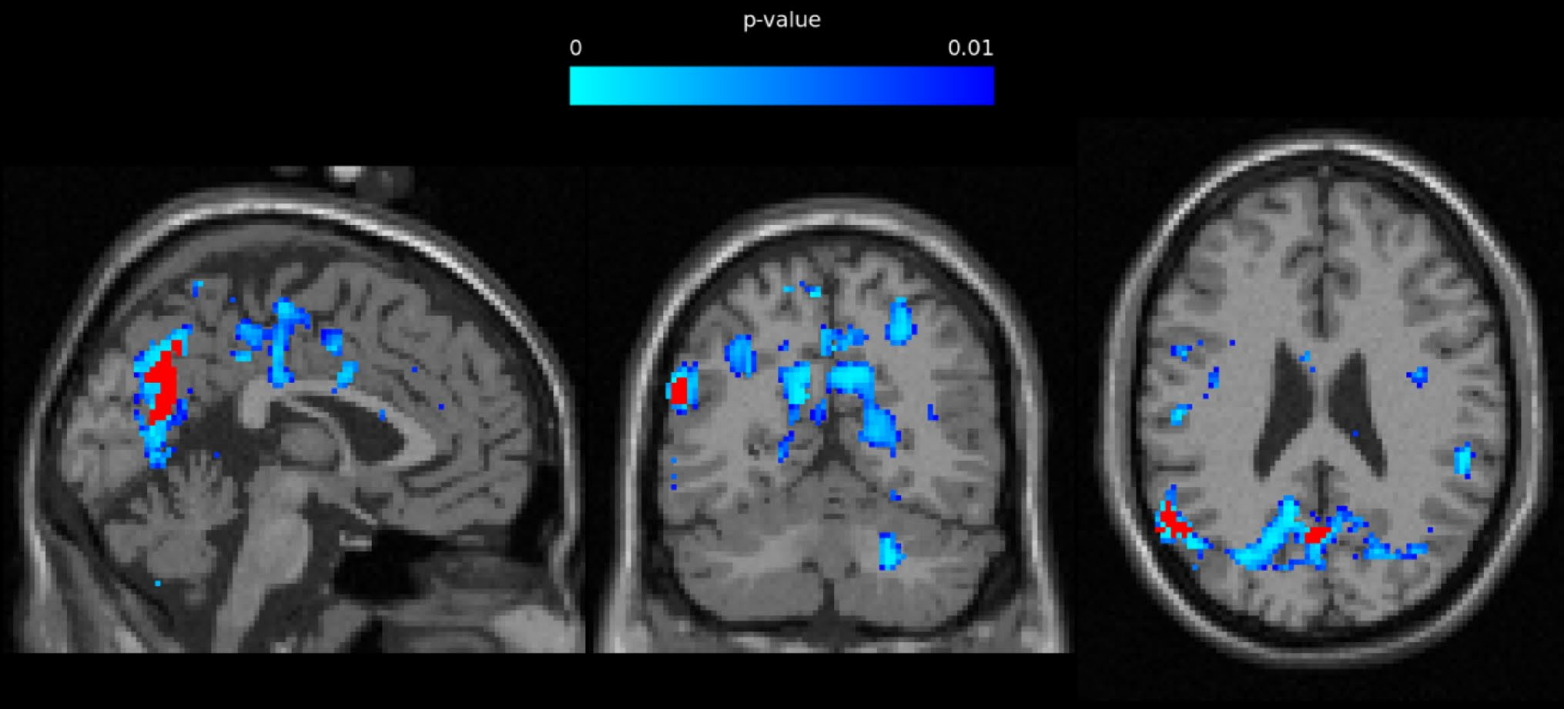
Choline group difference map. Voxels with significantly different choline (Cho/H_2_O) between groups are displayed in blue (*p* ≤ 0.01). Significantly different voxels between groups after adjusting for the FDR are displayed in red

Choline referenced to water (Cho/H_2_O) was significantly lower in GWI in left calcarine sulcus (*p* = 0.003),^1^ right calcarine sulcus (*p* = 0.001) (see footnote 1), left mid cingulate (*p* = 0.016), right mid cingulate (*p* = 0.001) (see footnote 1), left posterior cingulate (*p* = 0.031), right posterior cingulate (*p* = 0.036), left cuneus (*p* = 0.005) (see footnote 1), right cuneus (*p* = 0.002) (see footnote 1), left lingual gyrus (*p* = 0.020), right lingual gyrus (*p* = 0.011), left occipital (*p* = 0.037), right occipital gyrus (*p* = 0.014), left paracentral lobule (*p* = 0.013), right paracentral lobule (*p* = 0.033), left parietal lobe (*p* = 0.027), right parietal lobe (*p* = 0.024), left postcentral gyrus (*p* = 0.005) (see footnote 1), right precentral gyrus (*p* = 0.008) (see footnote 1), left precuneus (*p* = 0.005) (see footnote 1), right precuneus (*p* = 0.004) (see footnote 1), right Rolandic operculum (*p* = 0.040), left supplementary motor area (*p* = 0.027), and left temporal lobe (*p* = 0.023). In all significant regional group differences, GWI exhibited lower choline than HV. Voxel-based group differences can be seen in Fig. 1. Images were generated in the PRANA module in MIDAS. T1w and metabolite images were non-linearly transformed to MNI space and mean value images were calculated for each group. Spatially normalized *p* value maps were exported in NIFTI format, displayed in FSLeyes (Jenkinson et al. 2012) and overlayed on a reference MNI template.

Though ACB scores were not significantly different between groups (*U* = 477.50, *p* = 0.055), they were on average higher in GWI and ranged from 0 to 5 while the HV group scores ranged from 0 to 3. Four participants were removed from the GWI group so that the GWI (n = 16) and HV (n = 20) groups had equivalent ranges of 0 to 3. Average brain choline remained significantly lower in GWI in this truncated sample (*t*(34) = − 2.58, *p* = 0.014), and 20 of the 23 initially significant regions remained significantly lower in GWI. Left supplementary motor cortex (*t*(34) = − 1.98, *p* = 0.055), right paracentral lobule (*t*(34) = − 1.65, *p* = 0.12), and left occipital cortex (*t*(34) = − 1.96, *p* = 0.059) were no longer significantly different between groups, though an additional region, right temporal lobe, was significantly lower in GWI in this truncated sample (*t*(34) = − 2.22, *p* = 0.034).

#### N-acetyl aspartate

NAA/H_2_O was lower in GWI in 15 brain regions: left calcarine fissure (*p* = 0.003), left mid cingulate (*p* = 0.005), right mid cingulate (*p* = 0.007), right posterior cingulate (*p* = 0.036), left fusiform gyrus (*p* = 0.023), left hippocampus (*p* = 0.001), right insula (*p* = 0.049), right globus pallidus (*p* = 0.028), left parietal lobe (*p* = 0.050), left postcentral gyrus (*p* = 0.020), left precentral gyrus (*p* = 0.038), left Rolandic operculum (*p* = 0.011), right Rolandic operculum (*p* = 0.011), left temporal lobe (*p* = 0.006), and right temporal lobe (*p* = 0.036). There were no brain regions with significantly higher NAA/H_2_O in GWI.

To mitigate the potential effect of baseline differences in PTSD symptoms on NAA, a sensitivity analysis was performed by truncating the sample to remove PCL-5 score differences between groups. Seven participants were removed from the GWI group so that GWI and HV groups did not have significantly different PCL-5 scores (*U* = 221.50, *p* = 0.062). In this post-hoc analysis, average brain NAA was still significantly lower in GWI (*t*(31) = − 2.23, *p* = 0.034), and all regions remained significantly lower in GWI except for left precentral gyrus (*t*(31) = − 1.74, *p* = 0.093), and right temporal lobe (*t*(31) = − 1.56, *p* = 0.13). An additional four regions were found to have lower NAA in GWI; left posterior cingulate cortex (*t*(31) = − 2.07, *p* = 0.048), left parietal lobe (*t*(31) = − 2.68, *p* = 0.013), right calcarine fissure (*t*(31) = − 2.07, *p* = 0.047), and left cuneus (*t*(31) = − 2.21, *p* = 0.036).

#### Lactate

In reference to water, lactate (Lac/H_2_O) was significantly higher in GWI in the right anterior cingulate (*p* = 0.001) and left occipital lobe (*p* = 0.037). There were no brain regions with significantly lower lactate in GWI.

#### Myo-inositol

Referenced to water, myo-inositol (MI/H_2_O) was significantly lower in GWI in right mid cingulate (*p* = 0.036), right precentral gyrus (*p* = 0.015), and right temporal lobe (*p* = 0.041).

#### Brain temperature

One HV participant was excluded from brain temperature analyses due to physiologically impossible values (M = 49.22 °C/120.60 °F). The average apparent brain temperature for all remaining participants ranged from 35.77 °C/96.39 °F to 37.28 °C/99.10 °F. Brain temperature, calculated in reference to an amplitude weighted average of Cho, NAA, and Cre, was significantly different between groups in left calcarine fissure (*p* = 0.016), right cuneus (*p* = 0.042), left cuneus (*p* = 0.005), right occipital cortex (*p* = 0.045), left occipital cortex (*p* = 0.015), and left parietal cortex (*p* = 0.038). In all these regions, the GWI group exhibited higher average temperature. Averaged across the cortical lobes and cerebellum, apparent brain temperature was significantly higher in GWI (M = 36.83 °C/98.29 °F (SD = 0.33)) than HV (M = 36.62 °C/97.92 °F (SD = 0.32), *p* = 0.029).

### Arterial spin labeling

Thirty-eight complete ASL datasets were collected (18 HV, 20 GWI). After correcting for partial volume effects, the mean grey matter perfusion was 49.62 mL/100 g/min in HV and 46.76 mL/100 g/min in GWI. This group difference was not significant (*t*(36) = 1.08, *p* = 0.29).

### Exploratory analyses

#### Metabolites and demographic covariates

When demographic covariates were not included, average brain choline (*F*(1,34) = 10.24, *p* = 0.003), creatine (*F*(1,34) = 6.67, *p* = 0.015), and NAA (*F*(1,34) = 4.95, *p* = 0.033) were significantly different between groups, with pairwise comparisons indicating lower choline, NAA, and creatine in GWI. When age, BMI, ethnicity, and education were included as covariates, average brain choline (*F*(1,22) = 15.02, *p* < 0.001), NAA (*F*(1,22) = 5.15, *p* = 0.033), creatine (*F*(1,22) = 8.30, *p* = 0.009), and temperature (*F*(1,22) = 6.42, *p* = 0.019) were significantly different between groups. Average myo-inositol and lactate were not significantly different between groups. Post-hoc pairwise comparisons with Bonferroni corrections identified significantly higher temperature (*p* = 0.019) in the GWI group, and significantly lower choline (*p* < 0.001), NAA (*p* = 0.033), and creatine (*p* = 0.009) in GWI.

#### Metabolites and Gulf War exposures

Those in the GWI group reported significantly increased exposure to seeing destroyed enemy vehicles (*U* = 164.00, *p* = 0.025), coming into contact with destroyed enemy vehicles (*U* = 172.50, *p* = 0.042), sleeping less than 4 h in a 24 h period (*U* = 155, *p* = 0.007), seeing smoke from oil well fires (*U* = 179.50, *p* = 0.036), pesticides used on the skin (*U* = 179, *p* = 0.031), pesticides used on uniforms (*U* = 167.00, *p* = 0.010), pesticides sprayed in the area (*U* = 186.50, *p* = 0.036), receiving shots in the arm while in theater (*U* = 136.00, *p* = 0.007), and taking nerve agent protection pills (*U* = 164.50, *p* = 0.038).

There were no significant differences between groups in reported exposures to scud missiles exploding within one mile, being directly involved in ground combat, being directly involved in air combat, seeing American or allied troops that were badly wounded or killed, seeing Iraqis or civilians that had been badly wounded or killed, coming into contact with prisoners of war, seeing dead animals, coming into direct contact with dead animals, coming into contact with destroyed American vehicles hit by friendly fire, hearing chemical alarms, wearing a flea collar, or receiving shots in the buttocks while in theater.

Exploratory analyses assessing the relationship between possible cholinergic agent exposures (i.e., pesticides, hearing chemical alarms, and PB pills) and brain metabolites revealed lower average brain choline was associated with more frequent exposure to pesticides used on uniforms (ρ(39) = − 0.440, *p* = 0.005), pesticides used on the skin (ρ(39) = − 0.410, *p* = 0.010), and pesticides sprayed in the area (ρ(39) = − 0.428, *p* = 0.007). Greater exposure to pesticides used on uniforms was also associated with lower average brain NAA (ρ(39) = − 0.319, *p* = 0.048). Average creatine, lactate, MI, and brain temperature were not associated with any self-reported possible cholinergic agent exposures.

## Discussion

In this observational study, whole-brain MRS revealed several differences in metabolites between veterans with GWI and HV including lower choline, lower myo-inositol, lower NAA, higher lactate, and higher brain temperature. Lower choline was identified in GWI in 23 of the 47 brain regions tested. These differences were also related to self-reported Gulf War exposures, with the most significant relationship seen between lower choline and exposure to pesticides. These findings, though not conclusive, are the first to indicate a relationship between cholinergic agents and persistent cholinergic alterations in the brain in GWI more than 3 decades later.

There are several proposed mechanisms that may explain the relationship between cholinergic function and GWI. While a neuroinflammatory mechanism for GWI pathology was originally hypothesized, these findings do not reflect the typical MRS profile of neuroinflammation. In particular, neuroinflammation is usually associated with increased choline, as choline reflects a greater concentration and turnover of glia (Mader et al. 2008; Chang et al. 2013; Lind et al. 2021). Similarly, the glial marker myo-inositol (Hattingen et al. 2008) is expected to be elevated during neuroinflammation, but was found to be lower in three brain regions in GWI.

The findings suggest that alternative pathophysiological mechanisms should be explored. For example, in response to neurotoxin exposure, accumulation of acetylcholine in synapses following acetylcholinesterase inhibitor exposure can cause acute excitotoxicity and over-activation of cholinergic neurons that results in dysfunction of cholinergic pathways and secondary neuronal damage and loss (Chen 2012). As cholinergic signaling is essential for cognition and memory, damage to these pathways may partly contribute to nearly one-fifth of Gulf War veterans demonstrating mild cognitive impairment, generally at ages earlier than expected (Chao et al. 2024). Alterations in cholinergic signaling could also account for autonomic dysfunction observed in GWI (Haley et al. 2004; Jaquess et al. 2021) due to acetylcholine’s chief role in the autonomic nervous system. It is important to note, however, that ventral frontal regions of the brain were not well captured in this study due to lipid saturation band placement during the MRS scan and rejection of voxels due to poor quality. As the basal forebrain and brainstem have the most cholinergic activity, future research may consider specifically investigating these regions with single voxels studies.

Another possible consequence of dysfunctional cholinergic signaling is uninhibited cytokine production in the central nervous system (Rosas‐Ballina and Tracey 2009; Hoover 2017; Wu et al. 2021). In the cholinergic anti-inflammatory pathway (CAP), mediated by the vagus nerve, acetylcholine attenuates inflammatory immune responses by binding to α7 subunit receptors expressed on macrophages and inhibiting cytokine production (Rosas‐Ballina and Tracey 2009; Hoover 2017; Wu et al. 2021). Disruption of this pathway by cholinergic agents could have resulted in inflammation in GWI, which has been seen in blood assays of cytokines (Broderick et al. 2013; Parkitny et al. 2015; Janulewicz et al. 2019; Hodgin et al. 2022), acute phase proteins (James et al. 2019), and clinical blood counts (Johnson et al. 2013, 2016).

Further supporting the possibility of neurotoxicity in GWI, NAA was lower in GWI in cortical and subcortical regions. As NAA is confined to neurons, it serves as an indicator of neuronal health and integrity (Arnold et al. 2001; Rae 2014) and chronic inflammation (Mader et al. 2008). Our finding of lower NAA in right globus pallidus is consistent with prior findings of lower NAA in basal ganglia in GWI (Haley et al. 2000; Meyerhoff et al. 2001), and we additionally replicated lower NAA in left hippocampus as reported by Menon et al. (2004). We further observed lower NAA in 14 other brain regions that have not been previously tested in GWI, highlighting the advantage of a whole-brain MRS approach. In this sample, areas with significantly lower NAA tended to be temporal and medial-temporal structures suggesting neuronal loss in these regions, consistent with decreased cortical volumes seen in these areas in Gulf War veterans exposed to chemical warfare agents (Chao et al. 2010). Given these findings, future GWI research should explore the association between neurometabolite alterations and performance on cognitive measures.

As an astrocyte marker, myo-inositol is expected to increase in neuroinflammatory states due to gliosis and astrocytic reactivity (Hattingen et al. 2008). Consistent with this, myo-inositol is elevated in neurodegenerative conditions (Murray et al. 2014; Hirata et al. 2024; Madsen et al. 2024). Lower levels of myo-inositol, however, have been observed in hepatic encephalopathy related to glutamine accumulation and problems with cell volume homeostasis (Tran et al. 2020). Lower levels of myo-inositol have also been found in schizophrenia as a possible reflection of astrocytic dysfunction (Das et al. 2018). In the present study, myo-inositol was lower in three regions in GWI. As astrocytes help clear glutamate, astrocytic dysfunction may reduce extracellular glutamate clearance and result in redox imbalance and excitotoxic damage (Schousboe et al. 2014).

As organophosphates also induce damage through oxidative stress, the role of oxidative stress and mitochondrial injury have also been investigated in GWI. Because NAA is synthesized in mitochondria, regional decreases can also indicate mitochondrial dysfunction (Moffett et al. 2014). The widespread decreases in creatine seen in this sample are consistent with prior findings of ATP production impairment in GWI, thought to be due to mitochondrial damage by acetylcholinesterase inhibitor toxicity (Golomb et al. 2023, 2024). Due to creatine’s role in energy and ATP production, decreased creatine levels could reflect depleted energy storage or changes in brain energetics. As creatine’s concentration is greatest in glial cells, however, neuroinflammation is thought to be associated with elevations in creatine, though findings in neuroinflammatory disorders have been mixed (Chang et al. 2013; Ostojic et al. 2024).

Some findings in the present study support the neuroimmune hypothesis of GWI, namely elevated lactate in two brain regions and higher average brain temperature. Elevated brain lactate, a marker of anerobic metabolism, can reflect neuroinflammation (Albrecht et al. 2016), but may also reflect increased oxidative stress, hypoperfusion, or mitochondrial dysfunction (Bruhn et al. 1989; Lunsing et al. 2017). Some regions with greater temperature and elevated lactate, specifically bilateral cuneus and right anterior cingulate, have also demonstrated increased TSPO uptake on PET imaging in GWI (Alshelh et al. 2020), further suggesting elevations seen here are reflective of neuroinflammation in these areas. In this sample, elevated lactate in the cortex is not likely explained by cerebral hypoperfusion, as perfusion estimates were comparable between groups.

### Limitations

A primary limitation of the study is the small sample size and high number of statistical comparisons. Many regional findings were not significant with an FDR of 0.05. Confirmatory replication of these exploratory findings in larger samples is warranted. Another significant limitation to the study is that all Gulf War-era exposures were self-reported, and in this case, provided three decades following the conflict. Any associations between reported exposures and brain markers in the current results are purely exploratory.

Despite these widespread group differences in brain metabolites, baseline group differences besides GWI status could confound results. In this study, PTSD and depression scores were significantly higher in the GWI group than the HV group. Depression has been associated with elevated choline in MRS studies (Riley and Renshaw 2018), and therefore unlikely to account for the group differences observed here. PTSD, however, is often characterized on MRS by lower NAA in the cingulate cortex and hippocampus (Quadrelli et al. 2018; Swanberg et al. 2022), as seen in our GWI group. To screen for the effects of PTSD on the present results, sensitivity analyses demonstrated that the GWI group still showed significantly lower NAA even when groups had equivalent distributions on the PCL-5. Future studies with larger samples are recommended to explore the relationship between GWI, PTSD, and brain metabolites further.

## Conclusions

In this exploratory human observational study, GWI was associated with several metabolite alterations on whole-brain MRS. Compared to healthy Gulf War veterans, veterans with GWI showed widespread decreases in choline, NAA, and creatine, while lactate levels and apparent brain temperature were elevated in some regions. These findings are consistent with what has been observed in animals exposed to toxic levels of organophosphates, who show acute increases in brain choline followed by delayed decreases in choline, NAA, and increases in lactate in the late phase (7 days post-exposure) (Fauvelle et al. 2010; Shrot et al. 2012). These preliminary findings provide support for neuronal damage and cholinergic deficits in GWI decades following the Persian Gulf War. Decreases in choline, NAA, and creatine could all be theoretically tied to acetylcholinesterase inhibitor exposure causing alterations to cholinergic signaling pathways, neurons, and mitochondria, respectively. Off-target inflammatory effects of the cholinergic agents could account for the elevated brain temperature and lactate. Furthermore, neuronal damage, cholinergic deficits, and bioenergetic impairments could explain many of the cognitive, neurologic, and mood symptoms in GWI. Further effects of cholinergic alterations on autonomic function and systemic inflammation could account for many of the multisystem symptoms in GWI. Although results are exploratory, they warrant future investigation and highlight the utility of whole-brain MRS in studying this population.

## Supplementary Information

Below is the link to the electronic supplementary material.

Supplementary Material 1

Supplementary Material 2

Supplementary Material 3

## Data Availability

The data presented in this study are available from the corresponding author upon reasonable request.

## Footnotes

1 Significant with a false discovery rate of 5%.
