# Supplementary material for: Neurometabolite alterations in Gulf War Illness: a whole-brain magnetic resonance spectroscopy study
Source: Exp Brain Res. 2025 Oct 25;243(11):237. doi: 10.1007/s00221-025-07174-w (PMC12553559; doi:10.1007/s00221-025-07174-w)
Supplement: Supplementary file 3 — Supplementary Material 3 [file 221_2025_7174_MOESM3_ESM.docx]

*Title: Neurometabolite Alterations in Gulf War Illness: A Whole-Brain Magnetic Resonance Spectroscopy*

*Study Authors: Chloe Jones*, Olivia Haskin, Jarred Younger*

*Affiliation for all authors: Department of Psychology, The University of Alabama at Birmingham,  Birmingham, AL, USA 35233*

******Correspondence****:* [*chloej@uab.edu*](mailto:chloej@uab.edu)

*Nonparametric Bivariate Correlations between Average Brain Markers and Symptoms Controlling for Age and BMI*

| **Average Brain Choline Pairwise Correlations** | **Coefficient** | **Significance** | **FDR-Adjusted Significance** |
| --- | --- | --- | --- |
| Kansas Pain & Average Brain Choline | -0.47 | **<0.01** | **0.01** |
| FSS & Average Brain Choline | -0.43 | **0.01** | **0.02** |
| Kansas Fatigue & Average Brain Choline | -0.35 | **0.03** | 0.07 |
| Kansas Total Average & Average Brain Choline | -0.33 | **0.04** | 0.08 |
| PHQ-9 Sum & Average Brain Choline | -0.31 | 0.06 | 0.10 |
| Kansas Neurologic/Cognitive/Mood & Average Brain Choline | -0.31 | 0.06 | 0.11 |
| BPI-S & Average Brain Choline | -0.27 | 0.12 | 0.19 |
| Kansas Gastrointestinal & Average Brain Choline | -0.24 | 0.14 | 0.22 |
| Kansas Respiratory & Average Brain Choline | -0.24 | 0.15 | 0.24 |
| Kansas Skin & Average Brain Choline | -0.20 | 0.24 | 0.33 |
| PCL-5 Sum & Average Brain Choline | -0.19 | 0.27 | 0.36 |
| **Average Brain Creatine Pairwise Correlations** | **Coefficient** | **Significance** | **FDR-Adjusted Significance** |
| Kansas Pain & Average Brain Creatine | -0.43 | **0.01** | **0.02** |
| FSS & Average Brain Creatine | -0.40 | **0.02** | **0.03** |
| Kansas Gastrointestinal & Average Brain Creatine | -0.35 | **0.03** | 0.06 |
| BPI-I & Average Brain Creatine | -0.36 | **0.04** | 0.07 |
| Kansas Total Average & Average Brain Creatine | -0.33 | **0.04** | 0.08 |
| Kansas Neurologic/Cognitive/Mood & Average Brain Creatine | -0.30 | 0.07 | 0.12 |
| Kansas Fatigue & Average Brain Creatine | -0.29 | 0.07 | 0.12 |
| BPI-S & Average Brain Creatine | -0.19 | 0.27 | 0.35 |
| Kansas Skin & Average Brain Creatine | -0.19 | 0.27 | 0.36 |
| PHQ-9 Sum & Average Brain Creatine | -0.17 | 0.32 | 0.40 |
| PCL-5 Sum & Average Brain Creatine | -0.13 | 0.46 | 0.54 |
| Kansas Respiratory & Average Brain Creatine | -0.05 | 0.76 | 0.80 |
| **Average Brain Lactate Pairwise Correlations** | **Coefficient** | **Significance** | **FDR-Adjusted Significance** |
| Kansas Respiratory & Average Brain Lactate | 0.22 | 0.21 | 0.30 |
| PHQ-9 Sum & Average Brain Lactate | 0.20 | 0.23 | 0.33 |
| FSS & Average Brain Lactate | 0.17 | 0.34 | 0.42 |
| Kansas Skin & Average Brain Lactate | -0.17 | 0.34 | 0.42 |
| BPI-I & Average Brain Lactate | 0.16 | 0.37 | 0.45 |
| Kansas Fatigue & Average Brain Lactate | -0.12 | 0.51 | 0.59 |
| PCL-5 Sum & Average Brain Lactate | 0.10 | 0.58 | 0.66 |
| BPI-S & Average Brain Lactate | 0.07 | 0.70 | 0.76 |
| Kansas Neurologic/Cognitive/Mood & Average Brain Lactate | 0.06 | 0.72 | 0.77 |
| Kansas Gastrointestinal & Average Brain Lactate | 0.05 | 0.76 | 0.80 |
| Kansas Pain & Average Brain Lactate | -0.04 | 0.83 | 0.86 |
| Kansas Total Average & Average Brain Lactate | 0.03 | 0.87 | 0.89 |
| **Average Brain Myo-Inositol Pairwise Correlations** | **Coefficient** | **Significance** | **FDR-Adjusted Significance** |
| BPI-I & Average Brain Myo-Inositol | -0.25 | 0.15 | 0.23 |
| Kansas Total Average & Average Brain Myo-Inositol | -0.23 | 0.17 | 0.26 |
| FSS & Average Brain Myo-Inositol | -0.23 | 0.19 | 0.28 |
| Kansas Neurologic/Cognitive/Mood & Average Brain Myo-Inositol | -0.21 | 0.21 | 0.31 |
| BPI-S & Average Brain Myo-Inositol | -0.21 | 0.23 | 0.32 |
| Kansas Fatigue & Average Brain Myo-Inositol | -0.20 | 0.24 | 0.33 |
| Kansas Pain & Average Brain Myo-Inositol | -0.20 | 0.24 | 0.33 |
| PHQ-9 Sum & Average Brain Myo-Inositol | -0.17 | 0.34 | 0.42 |
| Kansas Gastrointestinal & Average Brain Myo-Inositol | -0.15 | 0.37 | 0.45 |
| PCL-5 Sum & Average Brain Myo-Inositol | -0.07 | 0.68 | 0.74 |
| Kansas Respiratory & Average Brain Myo-Inositol | -0.07 | 0.70 | 0.76 |
| Kansas Skin & Average Brain Myo-Inositol | 0.00 | 0.99 | 0.99 |
| **Average Brain NAA Pairwise Correlations** | **Coefficient** | **Significance** | **FDR-Adjusted Significance** |
| Kansas Total Average & Average Brain NAA | -0.35 | **0.03** | 0.06 |
| Kansas Pain & Average Brain NAA | -0.33 | **0.04** | 0.08 |
| FSS & Average Brain NAA | -0.33 | 0.05 | 0.09 |
| Kansas Neurologic/Cognitive/Mood & Average Brain NAA | -0.32 | 0.05 | 0.09 |
| Kansas Fatigue & Average Brain NAA | -0.30 | 0.06 | 0.11 |
| BPI-I & Average Brain NAA | -0.27 | 0.11 | 0.18 |
| Kansas Gastrointestinal & Average Brain NAA | -0.23 | 0.17 | 0.26 |
| PHQ-9 Sum & Average Brain NAA | -0.19 | 0.24 | 0.33 |
| BPI-S & Average Brain NAA | -0.15 | 0.39 | 0.47 |
| Kansas Respiratory & Average Brain NAA | -0.12 | 0.48 | 0.55 |
| Kansas Skin & Average Brain NAA | -0.11 | 0.51 | 0.59 |
| PCL-5 Sum & Average Brain NAA | -0.04 | 0.83 | 0.86 |
| **Average Brain Temperature Pairwise Correlations** | **Coefficient** | **Significance** | **FDR-Adjusted Significance** |
| BPI-I & Average Brain Temperature | 0.23 | 0.20 | 0.28 |
| PHQ-9 Sum & Average Brain Temperature | 0.20 | 0.24 | 0.33 |
| Kansas Total Average & Average Brain Temperature | 0.20 | 0.24 | 0.33 |
| Kansas Respiratory & Average Brain Temperature | 0.17 | 0.31 | 0.40 |
| Average Brain NAA & Average Brain Temperature | -0.17 | 0.31 | 0.40 |
| FSS & Average Brain Temperature | 0.14 | 0.43 | 0.51 |
| Kansas Neurologic/Cognitive/Mood & Average Brain Temperature | 0.13 | 0.45 | 0.54 |
| Kansas Skin & Average Brain Temperature | 0.11 | 0.52 | 0.59 |
| Kansas Pain & Average Brain Temperature | 0.09 | 0.59 | 0.66 |
| BPI-S & Average Brain Temperature | 0.09 | 0.61 | 0.67 |
| PCL-5 Sum & Average Brain Temperature | -0.03 | 0.85 | 0.88 |
| Average Brain Lactate & Average Brain Temperature | -0.01 | 0.97 | 0.98 |
